# Combining Controlled-Release and Normal Urea Enhances Rice Grain Quality and Starch Properties by Improving Carbohydrate Supply and Grain Filling

**DOI:** 10.3390/plants14010107

**Published:** 2025-01-02

**Authors:** Chang Liu, Tianyang Zhou, Zhangyi Xue, Chenhua Wei, Kuanyu Zhu, Miao Ye, Weiyang Zhang, Hao Zhang, Lijun Liu, Zhiqin Wang, Junfei Gu, Jianchang Yang

**Affiliations:** 1 Jiangsu Key Laboratory of Crop Genetics and Physiology, Jiangsu Key Laboratory of Crop Cultivation and Physiology, Agricultural College, Yangzhou University, Yangzhou 225009, China; mx120220734@stu.yzu.edu.cn (C.L.); dx120210114@stu.yzu.edu.cn (T.Z.); mx120200689@stu.yzu.edu.cn (C.W.); kyzhu@yzu.edu.cn (K.Z.); 007867@yzu.edu.cn (M.Y.); wyz@yzu.edu.cn (W.Z.); haozhang@yzu.edu.cn (H.Z.); ljliu@yzu.edu.cn (L.L.); zqw@yzu.edu.cn (Z.W.); 2 Jiangsu Co-Innovation Center for Modern Production Technology of Grain Crops, Yangzhou University, Yangzhou 225009, China

**Keywords:** controlled-release nitrogen fertilizer, yield, quality, rice (*Oryza sativa* L.), starch, structure and physicochemical properties

## Abstract

Controlled-release nitrogen fertilizers are gaining popularity in rice (*Oryza stavia* L.) cultivation for their ability to increase yields while reducing environmental impact. Grain filling is essential for both the yield and quality of rice. However, the impact of controlled-release nitrogen fertilizer on grain-filling characteristics, as well as the relationship between these characteristics and rice quality, remains unclear. This study aimed to identify key grain-filling characteristics influencing rice milling quality, appearance, cooking and eating qualities, and physicochemical properties of starch. In this study, a two-year field experiment was conducted that included four nitrogen management practices: zero nitrogen input (CK), a local high-yield practice with split urea applications (100% urea, CU), a single basal application of 100% controlled-release nitrogen fertilizer (CRNF), and a basal application blend of 70% controlled-release nitrogen fertilizer with 30% urea (CRNF-CU). The results showed that a sufficient amount of carbohydrates for the rice grain-filling process, as indicated by a higher sugar–spikelet ratio, is essential for improving grain quality. An increased sugar–spikelet ratio enhances the grain-filling process, resulting in an elevated average grain-filling rate (G_mean_) and the peak grain-filling rate (G_max_), while also reducing the overall time required for grain filling (D). Compared to CU, CRNF and CRNF-CU treatments did not significantly change milling qualities, but reduced the chalky kernel rate and chalkiness, thereby enhancing the appearance quality. These treatments increased the amylose and amylopectin contents while reducing protein content, though the proportion of protein constituents remained unchanged. These treatments led to larger starch granules with notably smoother surfaces. Additionally, CRNF and CRNF-CU reduced relative crystallinity and structural order, while increasing the amorphous structure in the outer region of starch granules, which lowered rice starch crystal stability. The treatments also increased viscosity and improved the thermodynamic properties of starch, resulting in enhanced eating and cooking quality of the rice. In conclusion, the CRNF-CU is the most effective strategy in this study to enhance both grain yield and quality. This practice ensures an adequate carbohydrate supply for grain filling, which is essential for efficient grain filling and improved overall quality.

## 1. Introduction

Modern agriculture faces significant challenges in ensuring global food security due to rising food demands and decreasing arable land. Past successes in crop production in China mainly relied on a ‘high input, high output’ approach [1]. However, excessive chemical fertilizer use, especially nitrogen (N), has led to eutrophication and pollution, negatively impacting environmental conditions and human health [2,3]. Due to high nitrogen fertilizer application rates, China’s current nitrogen recovery efficiency is only 25–35%—about 50% lower than in other leading regions—which is unsustainable [4].

The Chinese Ministry of Agriculture initiative launched the ‘Zero Increase Action Plan’ in 2015, aimed at halting the increase in fertilizer use by 2020 [5]. The goal was to improve crop yields while reducing the environmental harm caused by excessive fertilizer application. Recently, controlled-release nitrogen fertilizers (CRNF) are designed to gradually release nitrogen, optimizing nutrient availability for crops while reducing environmental losses. Commercial CRFs using polymer coatings are mostly made of thermoplastic resin such as polyolefin, polyvinylidene chloride, and copolymers. Nitrogen release from controlled-release fertilizers is influenced by temperature, moisture, and microorganisms [6,7]. Higher temperatures increase coating permeability and microbial activity, while adequate moisture aids diffusion. Excess water may cause leaching. Soil pH and irrigation practices also affect release, highlighting the need to synchronize nitrogen availability with crop demand for efficiency. They have been extensively applied to rice [6], wheat [7], maize [8], and other field crops [9]. CRNF can enhance nitrogen use efficiency (NUE), reduce the frequency of applications, and lower risks of nitrogen losses through leaching and ammonia volatilization [10]. Xu et al. [11] found that CRNF reduced ammonium concentrations in surface water and soil solution. Additionally, CRNF has been shown to improve root architecture [12], increase activities of nitrate reductase and glutamine synthetase [13], and maintain photosynthesis in late growth stages [14]. Furthermore, CRNF use in rice paddies has been associated with reduced greenhouse gas emissions, compared to conventional urea [10].

The gradual release of nitrogen from CRNF ensures a steady nutrient supply for plant uptake over the entire growing season. Consequently, using CRNF in rice production can enhance yield and improve nitrogen use efficiency [10]. Nonetheless, the release of nitrogen (N) from the polymer coating might be too gradual to provide an adequate supply of nitrogen during the initial growth phase of plants [15]. Recent studies have been focused on blending CRNF and CU (CRNF-CU) [6,16,17]. For example, CRNF-CU has been shown to increase nitrate content in the topsoil while lowering residual nitrate in deeper soil layers, reducing the potential for nitrogen leaching [18]. CRNF-CU offers an effective approach to improve nitrogen use efficiency (NUE) and minimize environmental impact, aligning well with China’s zero-growth fertilizer plan. This combination provides a consistent nitrogen supply, reducing nutrient losses through leaching and volatilization. Studies have demonstrated that CRNF-CU can significantly increase yields, economic returns, and NUE [18,19].

The process of grain filling is essential for determining rice yield and quality, and it is greatly affected by the use of fertilizers. Improper fertilizer application can adversely affect the appearance of rice grain, leading to chalkiness due to an imbalance in C–N metabolism [20,21]. In order to guarantee a sufficient amount of carbohydrates for the rice grain-filling process, it is essential to have an optimal level of nitrogen, which facilitates photosynthesis, promotes the transfer of carbon to the grains, grain filling, and supports the synthesis of related enzymes [21]. The availability of nitrogen significantly affects C-N metabolism, which in turn directs the production of a range of macromolecules such as starches, proteins, and lipids during grain filling. Undoubtedly, CRNF and CRNF-CU enhance crop yield by efficiently managing the soil’s nitrogen supply, thereby influencing the source–sink relationship and ultimately improving grain quality.

The primary components of a polished rice grain are starch (up to 95% dry weight) [22], and its structure and physicochemical properties are crucial for grain quality [20,23,24,25]. The synthesis of starch is rather complicated [26]. Starch formation begins with sucrose synthesis via photosynthesis, which is hydrolyzed by sucrose synthase (SUS) into fructose and UDP-glucose (UDPG), converted to ADP-glucose (ADPG) by ADP-glucose pyrophosphorylase (AGPase), and finally transformed into glucan through a series of enzymatic reactions [27]. Key enzymes in starch synthesis, including AGPase, soluble starch synthase (SS), granule-bound starch synthase (GBSS), starch branching enzyme (SBE), and starch debranching enzyme (DBE), also affect grain sink capacity and activity [21,25,28,29]. Key starch properties—such as granule size, amylose and amylopectin content, and amylopectin branch chain length—affect its crystallinity and stability, leading to changes in the starch’s physical and chemical characteristics and ultimately impacting its cooking and gelatinization properties [20,24]. Wei et al. [27] reported that an increased supply of photosynthetic assimilates and enhanced starch synthesis can lead to improved grain quality. The synthesis of starch relayed on C-N metabolism [21]. However, the starch synthesis and grain filling under the influence of CRNF and CRNF-CU are not yet fully understood.

Nitrogen fertilizer is the most essential nutrient for crop productivity and quality. Applying an appropriate amount of nitrogen fertilizer can increase crop productivity by increasing leaf development and photosynthesis. While higher amounts of nitrogen fertilizer can raise protein levels, it can also negatively impact the cooking and eating qualities of the grain, affecting starch granule size, chain length, crystal structure, and pasting properties [20,24,30,31]. Though the use of CRNF in rice cultivation is common for increasing grain production and N use efficiency, the impact on starch synthesis and structure, as well as the correlation between starch fine structure and rice quality, remains poorly comprehended. As living standards rise in China, there is a growing demand for high-quality rice, particularly in terms of its eating quality. To meet these holistic objectives—stable production, superior quality, decreased labor intensity, and environmental sustainability—enhanced fertilization practices will be crucial.

In this study, a two-year field experiment was conducted to examine the impact of CRNF and CRNF-CU on grain quality of rice. We studied rice appearance and milling quality, cooking and tasting quality, and the starch properties. We hope our study will contribute to improving rice quality and yield through the use of slow-controlled release fertilizers.

## 2. Results

### 2.1. Grain Yield and Its Components

**Figure 1:**
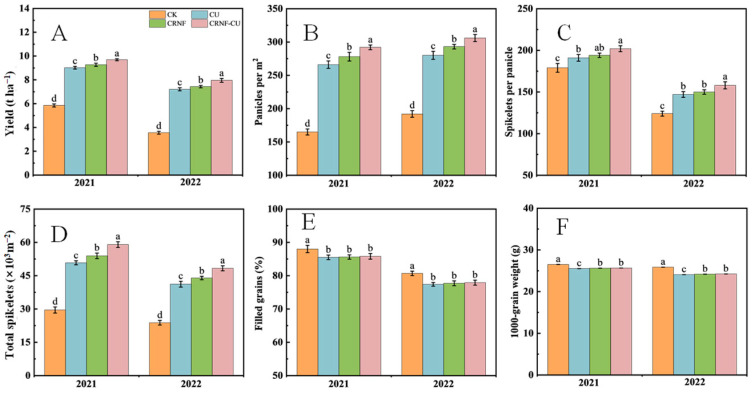
The effects of different nitrogen fertilizer treatments on grain yield (**A**) and its components (panicles per m^2^, (**B**); spikelets per panicle, (**C**); total sipkelets per m^2^, (**D**); filled grains, (**E**); 1000-grain weight, (**F**)). Different letters indicate significant differences between different treatments of the same year (*p* < 0.05). CK, control check treatment; CU, conventional urea treatment; CRNF, controlled-release nitrogen fertilizer treatment; CRNF-CU, combined 30% conventional urea with 70% controlled-release nitrogen fertilizer treatment.

Figure 1 presents the grain yield and its components. Without nitrogen fertilizer application, the CK treatment showed the lowest grain yield and total spikelet number per area, yet it achieved the highest ratio of filled grains and the greatest 1000-grain weight. Nitrogen fertilizer application significantly boosted grain yield and affected yield components, with variations observed between different fertilizer types. The CRNF and CRNF-CU treatments increased yield by an average of 2.95% and 8.93%, respectively, over two consecutive years compared to the CU treatment. Regarding yield components, controlled-release nitrogen fertilizer, relative to CU, increased the number of panicles, spikelets per panicle, total spikelets, and 1000-grain weight. Specifically, total spikelet number increased by 9.71% with the CRNF treatment and by 14.31% with the CRNF-CU treatment. The increase in spikelet number was the most substantial factor contributing to yield enhancement. In this study, we adopted a nitrogen fertilization rate of 240 kg N ha^−1^ to align with the current high-yielding farmers’ practice in the study region. While this choice provides practical relevance, it is important to acknowledge that such a level of nitrogen input is disproportionately high and unsustainable, as highlighted by Cai et al. [4]. These findings underscore the pressing need for targeted interventions to enhance NUE and reduce excessive nitrogen applications in rice systems, thereby improving both productivity and environmental sustainability. Our results should not be interpreted as justifying the current high levels of nitrogen input but rather as a call to action for developing and adopting sustainable nitrogen management practices. In this study, we combined CRNF and CU, which demonstrated improvements in yield, NUE, and grain quality. Further research and practical applications are needed to refine these strategies, lower nitrogen inputs, and promote sustainable nitrogen management.

### 2.2. Grain-Filling Characteristics and the Sugar–Spikelet Ratio

**Figure 2:**
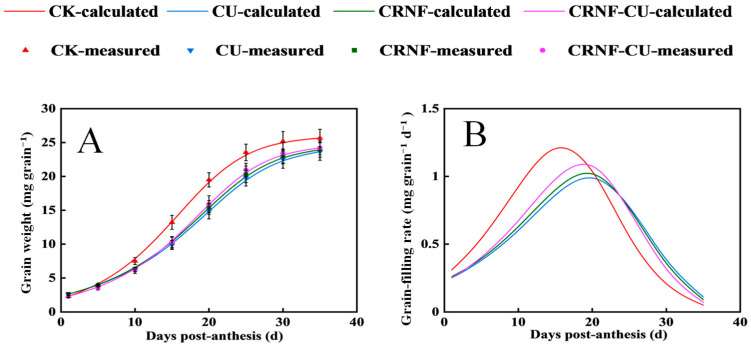
The effects of different nitrogen fertilizer treatments on the dynamics of grain weight (**A**) and the dynamics of grain-filling rate (**B**). Both measured values and fitted curves are plotted for grain weight changes, while only calculated curves are shown for grain-filling dynamics. CK, control check treatment; CU, conventional urea treatment; CRNF, controlled-release nitrogen fertilizer treatment; CRNF-CU, combined 30% conventional urea with 70% controlled-release nitrogen fertilizer treatment.

The dynamics of grain filling are presented in Figure 2A. By fitting the Richards model, the parameters of maximum grain-filling rate (G_max_), mean grain-filling rate (G_mean_), and active grain-filling period (D) are calculated (Table 1). The dynamic grain-filling rate are presented in Figure 2B.

The CK treatment showed the highest G_max_ and G_mean_, reaching its peak grain-filling rate earliest and having the shortest active grain-filling period. Compared to the CU treatment, the use of controlled-release nitrogen fertilizer shortened the active grain-filling period but increased the grain-filling rate. Specifically, for the CRNF-CU treatment, G_mean_ and G_max_ were 7.58% and 6.73% higher than those in CU, respectively. Additionally, CRNF and CRNF-CU significantly increased grain weight, with the CRNF-CU treatment showing the most pronounced effect.

The sugar–spikelet ratio refers to the proportion of non-structural carbohydrates (NSCs) in the stems relative to the overall number of spikelets at heading. A higher sugar–spikelet ratio at heading indicates more NSCs stored in vegetative tissues relative to the number of spikelets. The CK treatment showed the highest sugar–spikelet ratio. Compared to the CU treatment, the sugar–spikelet ratio was 2.96% higher in the CRNF treatment and 6.27% higher in the CRNF-CU treatment. These results indicated that the application of controlled-release nitrogen fertilizer can improve the grain-filling characteristics of rice and the sugar–spikelet ratio.

### 2.3. Rice Appearance Quality and Milling Quality

The appearance and milling qualities of different treatments are shown in Table 2. The CK treatment exhibited the best appearance quality, characterized by the lowest chalkiness and chalky kernel rate. However, it showed the poorest milling quality, with significantly reduced brown rice rate, milled rice rate, and head milled rice rate compared to other treatments. The application of nitrogen fertilizer significantly increased both chalky kernel rate and chalkiness. Notably, the chalky kernel rate in the CRNF and CRNF-CU treatments decreased by 7.04% and 7.87%, respectively, while chalkiness was reduced by 7.85% and 9.42%, respectively, compared to the CU treatment. There were no significant differences in milling qualities among the CU, CRNF, and CRNF-CU treatments.

### 2.4. The Contents of Amylose, Amylopectin, Protein, and Rice Eating Qualities

The contents of amylose, amylopectin, protein, and rice eating qualities of different treatments are showed in Table 3. Among all the treatments, the highest levels of amylose and amylopectin, along with the lowest protein content were observed in CK treatment. Among the nitrogen application treatments, the use of controlled-release nitrogen fertilizer increased the amylose and amylopectin content and reduced the protein content compared to the CU treatment, though these changes were no statistical difference. These results may be attributed to the equivalent amounts of nitrogen applied across the CU, CRNF, and CRNF-CU treatments. Table 3 shows that the CK treatment had superior eating quality, featuring the highest gel consistency, viscosity, and taste value, along with the lowest hardness, all of which better match consumer preferences. Nitrogen fertilizer significantly deteriorated the eating quality. However, compared to the CU treatment, the gel consistency, viscosity, and taste value increased by 1.36% and 1.94%, 8.74% and 10.64%, and 9.22% and 10.73% for the CRNF and CRNF-CU treatments, respectively.

### 2.5. Starch Granule Morphology

**Figure 3:**
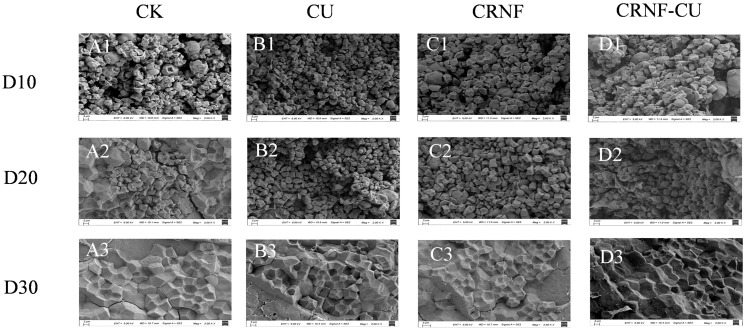
The effects of different nitrogen fertilizer treatments on the morphology of rice starch granules in 2022. (**A**–**D**) The morphology of rice starch granules of grain filling under CK, CU, CRNF, and CRNF-CU treatments, respectively; D10, D20, D30, the morphology of rice starch granules observed at 10, 20, and 30 days post anthesis, respectively. Magnifications = 2000×. CK, control check treatment; CU, conventional urea treatment; CRNF, controlled-release nitrogen fertilizer treatment; CRNF-CU, combined 30% conventional urea with 70% controlled-release nitrogen fertilizer treatment.

In all treatments, the starch granules displayed an irregular polygonal shape (Figure 3). Compared to CK, the application of nitrogen fertilizer significantly influenced the appearance and morphology. In the CK treatment, starch granules exhibited a smoother surface and larger size, whereas with nitrogen fertilizer application, the granule surfaces became uneven, and their size decreased. When comparing CRNF and CRNF-CU with CU, these starch granules’ surfaces were smoother and flatter and the dents on the surface of starch were decreased, the starch granules were arranged more closely, and the gaps were smaller. The CRNF-CU treatment was found to be the most effective in enhancing the shape and structure of starch granules when compared to the CRNF and CU treatments.

### 2.6. X-Ray Diffraction (XRD) and Relative Crystallinity

**Figure 4:**
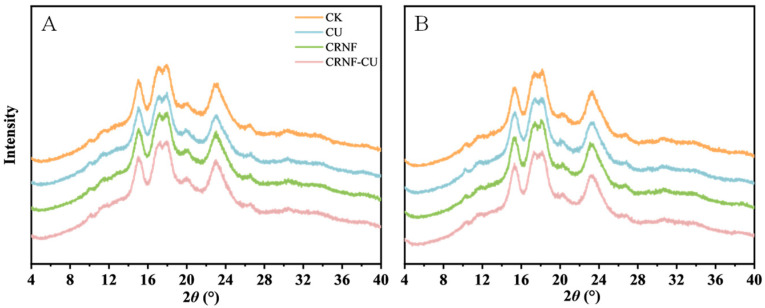
The effects of different nitrogen fertilizer treatments on the X-ray diffraction patterns of rice starch in 2021 (**A**) and 2022 (**B**). CK, control check treatment; CU, conventional urea treatment; CRNF, controlled-release nitrogen fertilizer treatment; CRNF-CU, combined 30% conventional urea with 70% controlled-release nitrogen fertilizer treatment.

XRD spectra indicated that rice starch exhibits an A-type pattern (Figure 4), with prominent diffraction peaks at 15° and 23°, along with an unresolved doublet at 17° and 18°. The effects of various nitrogen fertilizer treatments on the starch crystal structure of rice are shown in Table 4. The application of nitrogen fertilizer increased the relative crystallinity, with the CK treatment showing the lowest crystallinity. Compared to the CU, the relative crystallinity in the CRNF and CRNF-CU treatments showed a downward trend, decreasing by 1.36% and 1.94%, respectively.

### 2.7. Structural Order of the Starch External Region

**Figure 5:**
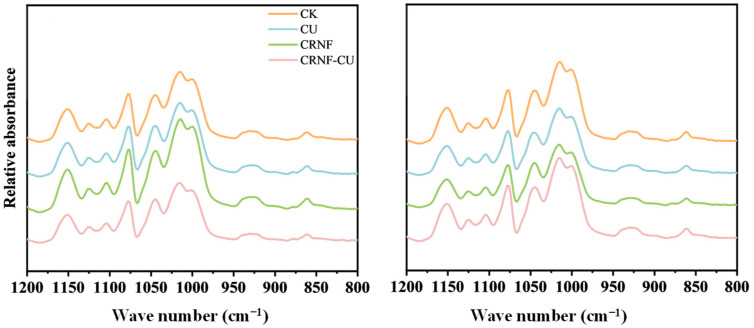
The effects of different nitrogen fertilizer treatments on starch Fourier Transform Infrared (FTIR) spectra of rice starch. CK, control check treatment; CU, conventional urea treatment; CRNF, controlled-release nitrogen fertilizer treatment; CRNF-CU, combined 30% conventional urea with 70% controlled-release nitrogen fertilizer treatment.

Fourier Transform Infrared (FTIR) spectroscopy is used to analyze the structural characteristics of starch (Figure 5). In FTIR, the absorption band at 1045 cm^−1^ is characteristic of the crystalline regions of starch, while the band at 1022 cm^−1^ is sensitive to the amorphous regions within starch granules. The ratio of the intensities of these two bands (1045/1022 cm^−1^) serves as an indicator of the degree of order in the external structure of starch. A higher ratio suggests a greater proportion of crystalline regions relative to amorphous regions, indicating a higher degree of order in the starch. Additionally, the 1022/995 cm^−1^ ratio is related to the proportion of amorphous regions to ordered carbohydrate structures in starch. Compared to CU, the application of controlled-release nitrogen fertilizer in the CRNF and CRNF-CU treatments increased the 1022/995 cm^−1^ ratio and decreased the 1045/1022 cm^−1^ ratio, with CRNF-CU showing the highest intensity at 1022/995 cm^−1^. CRNF and CRNF-CU treatments reduced starch stability.

### 2.8. Pasting Characteristics of Starch

The RVA (Rapid Viscosity Analyzer) characteristics refer to the viscosity properties of starch, which are key physical indicators of rice gelatinization characteristics and closely related to cooking and taste quality (Table 5). The CK treatment exhibited the highest values for peak viscosity, hot viscosity, breakdown, and final viscosity, along with the lowest values for setback and pasting temperature. Compared to CU, the CRNF and CRNF-CU treatments showed higher viscosity and breakdown values, along with a lower setback value. Additionally, both treatments reduced the pasting temperature. These results suggest that applying controlled-release nitrogen fertilizer may enhance rice quality by increasing viscosity and lowering the pasting temperature.

### 2.9. Thermal Properties of Starch

Table 6 presents the thermal properties of starch of different treatments. Significant differences in the thermal properties of starch were observed under various treatments. Gelatinization temperature refers to the temperature at which starch granules begin to swell, absorb water, and break down, resulting in a gel-like consistency. The temperature is crucial in food processing, as it affects the texture, viscosity, and digestibility of starchy foods. In general, rice with a higher gelatinization temperature requires more heat to start swelling and breaking down its starch granules. This typically results in firmer, less sticky rice upon cooking, as the starch resists gelatinization at lower temperatures. Rice grown under the CK treatment exhibited the lowest gelatinization temperature. Compared to CU, the CRNF and CRNF-CU treatments resulted in lower onset temperature (*T*_O_), peak temperature (*T*_P_), conclusion temperature (*T*_C_), and gelatinization enthalpy (Δ*H*_gel_), with CRNF-CU showing the lowest values.

### 2.10. Amino Acid Content in Rice Grain

**Figure 6:**
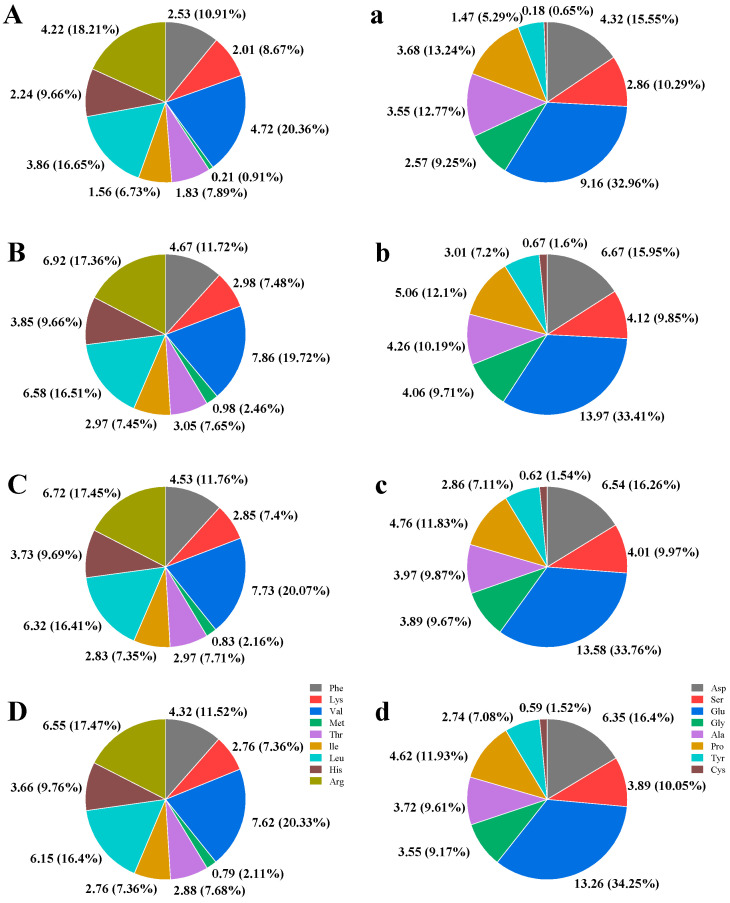
The essential amino acid content of rice grain (**A**–**D**) and non-essential amino acid content of rice grain (**a**–**d**) under CK (**A**,**a**), CU (**B**,**b**), CRNF (**C**,**c**), and CRNF-CU (**D**,**d**) treatments. Phe, phenylalanine; Lys, lysine; Val, valine; Met, methionine; Thr, threonine; Ile, isoleucine; Leu, leucine; His, histidine; Arg, arginase; Asp, aspartic; Ser, serine; Glu, glutamic; Gly, glycine; Ala, alanine; Pro, proline; Tyr, tyrosine; Cys, cysteine; CK, control check treatment; CU, conventional urea treatment; CRNF, controlled-release nitrogen fertilizer treatment; CRNF-CU, combined 30% conventional urea with 70% controlled-release nitrogen fertilizer treatment.

The contents of essential and non-essential amino acids in rice grain under different nitrogen treatments are presented in Figure 6. The content of valine was the highest, while the content of methionine was the lowest in essential amino acids. The content of glutamic was highest, while the content of cysteine was lowest in non-essential amino acids. Nitrogen fertilization increased the contents of amino acids significantly when compared with CK treatment. Compared to the CU treatment, the contents of essential and non-essential amino acids decreased under the CRNF and CRNF-CU treatments, but their relative proportions remained largely unchanged.

### 2.11. Correlations Between Grain-Filling-Related Traits and Grain Qualities

**Figure 7:**
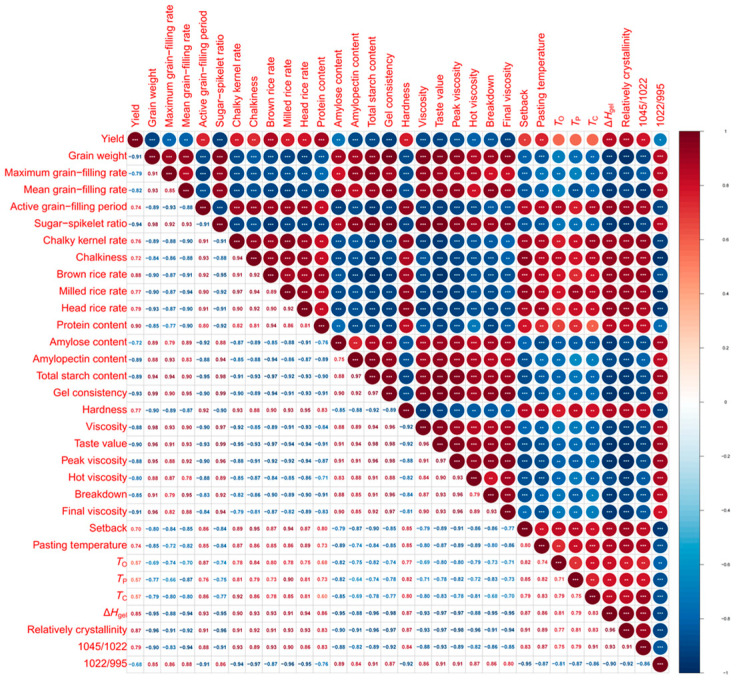
Heat map of correlations among traits of starch properties, grain filling and rice qualities. For analysis of variance, *, **, and *** significant differences at *p* < 0.05, *p* < 0.01, and *p* < 0.001, respectively.

**Figure 8:**
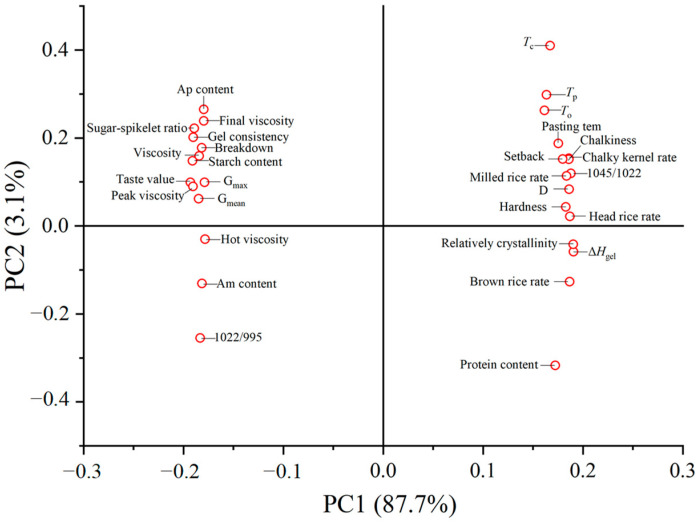
Principal component analysis (PCA) of traits of starch properties, grain filling and rice qualities. Am, amylose; Ap, amylopectin; *T*_O_, onset temperature; *T*_P_, peak temperature; *T*_C_, conclusion temperature; Δ*H*_gel_, gelatinization enthalpy; G_max_, maximum grain-filling rate; G_mean_, mean grain-filling rate; D, active grain-filling period.

The result of correlation analysis between grain-filling characteristics and rice qualities is shown in Figure 7. Sugar–spikelet ratio was significantly positively correlated with grain weight, G_max_, G_mean_, gel consistency, viscosity, and taste value, while G_max_ and G_mean_ were negatively correlated with chalky kernel rate, chalkiness hardness, and relatively crystallinity. Relatively crystallinity was negatively correlated with peak viscosity, hot viscosity breakdown, and final viscosity, and was positively correlated with setback pasting temperature, *T*_O_, *T*_P_, *T*_C_, and Δ*H*_gel_. A high sugar-flower ratio can enhance grain-filling rate, improve rice appearance and eating quality, reduce relative crystallinity, and enhance starch pasting and thermal properties. Figure 8 presents a principal component analysis illustrating the relationship between grain-filling characteristics and rice quality in Figure 7. The loading plot revealed that there was a strong correlation between the sugar–spikelet ratio and gel consistency; similarly, a close relationship was observed among G_max_, G_mean_, taste value, and peak viscosity; additionally, a tight association was found between setback, chalkiness, and the chalk kernel rate. The negative correlations also indicated that the contents of amylose and amylopectin were negatively associated with relative crystallinity and gelatinization enthalpy.

## 3. Discussion

Studies have demonstrated that the application of controlled-release nitrogen fertilizer can enhance rice production by increasing the number of panicles, the grains per panicle, and the weight of 1000 grains, consequently leading to higher yields [10]. In this experiment, both CRNF and CRNF-CU treatments increased rice yield compared to CU, primarily due to a higher total spikelet number, which aligns with findings from previous studies. The coordination of the release of controlled-release nitrogen fertilizer with the nitrogen demand period of rice can lead to increased yields [12]. This synchronization ensures that the rice plants receive the necessary nitrogen at the optimal times for growth and development, which can improve the efficiency of nitrogen use and contribute to higher grain production [10]. Compared with CRNF, CRNF-CU released a large amount of nitrogen after sowing, which promoted an increase in nitrogen absorption, tillering, leaf photosynthetic potential, and crop growth rate.

Rice quality is closely connected to the grain-filling process, and duration and rate of this process play a pivotal role in the final yield and quality [21,32]. In this experiment, sugar–spikelet ratio and grain-filling rate were tightly correlated. The sugar–spikelet ratio reflects the availability of soluble sugars, primarily sucrose, relative to the number of developing spikelets. This ratio indicates the balance between carbohydrate supply (sugars produced through photosynthesis) and demand (by spikelets during grain filling) [33]. A higher sugar–spikelet ratio generally supports better grain filling, as more assimilates are available to be transported into the grains. NSCs not only acts as an assimilate for grain filling, but also regulates sink activity at the early stage of grain filling, which plays an important role in promoting endosperm development and grain filling [29]. Wei et al. [27] also reported that source strength and sink size regulated expression of starch metabolic genes in grains which would change the structure and physicochemical properties of starch. In this study, the improved grain filling, as indicated by higher G_max_ and G_mean_, is closely related to enhanced appearance qualities and eating qualities. The application of controlled-release nitrogen fertilizer was found to regulate the accumulation of NSCs prior to the heading, thereby affecting the sugar–spikelet ratio. This regulation can lead to enhanced grain filling and a subsequent improvement in the overall quality of the grain. Our experiment also observed that G_max_ and G_mean_ were significantly and negatively correlated with protein content and hardness. These correlations underscore the direct impact of grain filling on rice quality, influencing the pasting properties and thermodynamic characteristics of starch, which are critical determinants of the eating and cooking quality of rice. In this study, the combined application of CU and CRNF was most effective in regulating the grain-filling process, resulting in optimal grain quality.

Chalkiness is an undesirable trait in rice appearance, mainly caused by insufficient filling of the amyloplast [34]. In chalky rice, starch granules are loosely packed with air pockets between them, scattering light and preventing its transmission, which creates a chalky appearance. The lowest chalkiness was observed in the CK treatment, likely due to a smaller sink size, higher accumulation of non-structural carbohydrates (NSCs), and a relatively higher sugar-to-spikelet ratio, all of which contributed to improved grain filling. In CU treatment, the lower sugar–spikelet ratio and slow grain-filling rate may be the reason of loose arrangement of starch granules, and ultimately increase the chalkiness rate of rice. The application of controlled-release nitrogen fertilizer could improve the grain filling, thereby reducing the chalkiness of milled rice. For the milling quality, in CK treatment, the sufficient filling of the amyloplast and larger seed size would increase fragility during milling, which could be the reason for the lower head rice rate. However, there was no significant difference in processing qualities among the treatments of different nitrogen fertilizer application.

Rice eating and cooking quality is influenced by multiple factors, such as amylose and protein content, gel consistency, and starch viscosity [35]. Studies have shown that rice with higher contents of protein and amylose generally has a firm texture, low viscosity, and poor taste [36,37]. Increased protein and amylose levels enhance the stability and heat resistance of the starch crystal structure, which restricts starch expansion and leaching during cooking, and resulting in a harder, less sticky rice texture [38]. In this experiment, compared with CU, the protein content decreased and the amylose content increased under CRNF and CRNF-CU treatments. The top-dressing of nitrogen fertilizer around heading time in CU treatment would increase protein content. The increase in protein content will make the rice texture harder, and the gel consistency and viscosity will decrease.

Starch accounts for about 90% of the dry weight of milled rice, and its properties significantly influence the grain quality [39]. Starch granules are composed of crystalline region and amorphous region. The crystalline regions are composed of tightly packed starch molecules in a highly ordered double-helix structure formed by the intertwining of amylopectin side chains. While the amorphous region is composed of amylose and partially ordered amylopectin, where the starch molecules are less ordered and more loosely arranged [40]. FTIR spectroscopy is a commonly employed analytical technique for examining structural properties of starch. Specifically, the peak intensity ratios at 1045/1022 cm^−1^ and 995/1022 cm^−1^ serve as quantitative indicators of the short-range molecular order in the peripheral region of the starch granule [20]. Our results indicated that applying CRNF, compared to CU, reduced the relative crystallinity of starch. Notably, the 1045/1022 cm^−1^ ratio was significantly lower in the CRNF and CRNF-CU treatments, indicating a reduced presence of ordered structures in the outer region of the starch granules.

The cooking and eating quality of rice is closely linked to starch pasting characteristics, showing a positive correlation with peak viscosity and breakdown value and a negative correlation with setback value. Gelatinization enthalpy, a measure of the energy required for starch gelatinization, reflects the loss of crystallinity and molecular order within the starch granules during heating. A higher gelatinization enthalpy indicates that more energy and a higher temperature are necessary to disrupt the double-helix structure [41]. In this experiment, for the application controlled-release nitrogen fertilizer, starch exhibited reduced crystallinity, which made starch perform better in starch pasting characteristics and thermal properties. The peak viscosity and breakdown value of starch were increased, and the setback value, gelatinization enthalpy, and gelatinization temperature were significantly reduced. It is reported that an increase in the short chains of amylopectin leads to a lower gelatinization temperature, and rice with a lower average chain length of amylopectin possesses superior pasting characteristics [42]. Concurrently, an increase in long-chain amylopectin branches forms more double-helix structures, leading to a tighter arrangement of starch granules and a more stable crystal structure [43]. The effect of controlled-release nitrogen fertilizer on the fine structure of rice still needs further study.

## 4. Materials and Methods

### 4.1. Experiment Location and Plant Material

**Figure 9:**
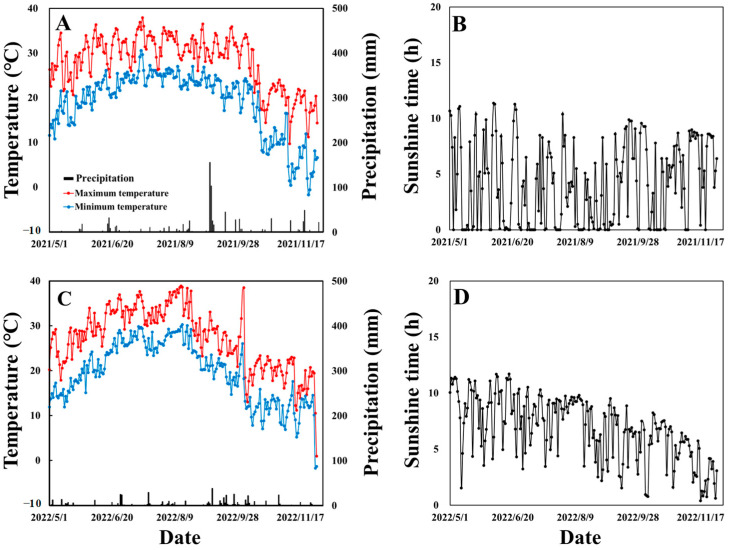
Daily maximum and minimum temperature (**A**,**C**), sunshine hours (**B**,**D**), and precipitation (A and C) during the rice growth stage in 2021 (**A**,**B**) and 2022 (**C**,**D**).

The experiments were conducted in 2021 and 2022 in Yangzhou, China (32°35′ N, 119°42′ E). The region belongs to a subtropical monsoon climate with humid changeable wind. The farming system in this region follows a rice–wheat rotation, with wheat being the previous crop. After wheat harvest, the straw and stubble were manually removed from the field, and the land was then evenly tilled using a rotary cultivator. The soil texture was sandy loam, containing 24.5 g·kg^−1^ organic matter, 108 mg·kg^−1^ alkali-hydrolyzed nitrogen, 68.5 mg·kg^−1^ available potassium, and 34.5 mg·kg^−1^ available phosphorus. The cultivar was Jinxiangyu1 (JXY-1), a *japonica* rice cultivar widely cultivated in Jiangsu Province. It was developed by the Lixiahe Agricultural Institute in Yangzhou, Jiangsu, China. JXY-1 has a growth duration of approximately 148 days and a plant height of around 96 cm. The variety exhibits moderate resistance to rice blast, bacterial leaf blight, and striped leaf blight, but is susceptible to narrow brown leaf spot. In both the experiment years, rice seeds were pre-germinated and sown in a seedbed between 22 and 25 May. The 25-day-old seedlings were then transplanted into a paddy field with a hill spacing of 0.107 m × 0.30 m, with two seedlings per hill. For weed control, both chemical (butachlor) and manual methods were employed. The pesticides Jinganmycin, imidacloprid, buprofezin, and triazophos were used to effectively manage pests and diseases. Detailed application methods are provided in the study of Qiu et al. [44]. The control of diseases, pests, and weeds were carried out following local high-yield practices. Meteorological data for daily maximum and minimum temperature, sunshine hours, and precipitation during the 2021 and 2022 rice-growing period were collected at a weather station near the experimental site (Figure 9).

### 4.2. Experiment Design and Treatments

There were four different nitrogen fertilizer treatments. The experimental setup was a **r**andomized block design with four treatments and three replications. Each plot had an area of 20 m^2^, and ridges were constructed between plots to prevent cross-contamination of fertilizers and water irrigation. Phosphorus (90 kg ha^−1^ as superphosphate) and 60% potassium (120 kg ha^−1^ as K_2_O) were applied and incorporated as a basal fertilizer before transplanting. The other potassium was applied during the panicle initiation stage. The experimental field was managed using alternate wetting and moderate drying (AWMD), allowing the shallow water layer to dry naturally until reaching a target water potential of −15 kPa at a depth of 15–20 cm below the surface, after which it was reflooded with a 1–2 cm water layer.

Four treatments were applied in the study. In the control (CK), no nitrogen fertilizer was used. In the conventional urea (CU) treatment, 240 kg N ha^−1^ was applied, with urea distributed in a 4:2:2:2 ratio across four stages: pre-transplanting, early tillering, panicle initiation, and spikelet differentiation. In the controlled-release nitrogen fertilizer (CRNF) treatment, 240 kg N ha^−1^ of CRNF was applied once as a basal fertilizer at pre-transplanting. In the combined CRNF-CU treatment, 240 kg N ha^−1^ was applied with a 3:7 ratio of conventional urea to CRNF, as a basal fertilizer at pre-transplanting.

### 4.3. Measurement of Grain Quality, Structure, and Physicochemical Properties of Starch

At the maturity stage, ten hills of rice plants in each plot were harvested to investigate the yield components. The brown rice rate, milled rice rate, head rice rate, kernel length to breadth, chalky grain rate, chalkiness, amylose content, protein content, and gel consistency are determined according to GB/T 17891-2017 [45].

The method of Richards [46] was used to evaluate the process of grain filling as the derivative of Equation (1):

(1) $$W=\frac{A}{{\left(1+Be^{-kt}\right)}^{1/N}}$$

where *W* represents grain weight, *A* is the maximum grain weight, *t* is the time after anthesis (d). The coefficients *B*, *k*, and *N* are determined by curve fitting. *e* is 2.71828, Euler’s number. Grain-filling rate (*R*) was calculated as the derivative of Equation (2):

(2) $$R=\frac{AkBe^{-kt}}{{N(1+Be^{-kt})}^{(N+1)/N}}$$

The active grain-filling period (D) was defined as the duration in which *W* ranges from 5% (*t*_1_) to 95% (*t*_2_) of *A*, with the average grain-filling rate calculated over this interval [29].

The non-structural carbohydrates (NSCs) were investigated according to the method of [47].

The taste value was determined by a taste analyzer (RCTA 11A, Satake Co., Hiroshima, Japan). A total of 30 g samples of head rice were cooked with a rice to water ratio of 1:1.3; after soaking, steaming, and air-cooling, 8 g cooked rice was pressed with a matching iron ring to determine the taste value. Texture properties were measured according to the method of Zhou et al. [20]. The amino acid content of rice grain was measured following the method of Mossé et al. [48] with an automatic amino acid analyzer (Biochrom 30, Biochrom, Cambridge, UK).

We used the neutral protease to remove the protein and to extract the starch from samples according to the method of Tran [49]. X-ray diffraction (XRD) and Fourier Transform Infrared (FTIR) were measured according to the method of Zhou et al. [24]. Rice paste properties were determined using a Rapid Visco Analyzer (RVA-3D, Newport Scientific, Warriewood, Australia) according to the method of Wei et al. [27]. Thermal properties were investigated by differential scanning calorimetry (DSC 200 F3, Netzsch, Selb, Germany) according to the method of Zhou et al. [20]. For the starch granule morphology observation, samples were collected at 10, 20 and 30 days post anthesis. The samples were observed and then photographed using scanning electron microscopy (Zeiss, SEM Gemini300, Oberkochen, Germany).

### 4.4. Grain Yield and Yield Components

Plants were harvested on 18–20 October in both of the two years. Grain yield and yield components were determined from a harvest area of 6.0 m^2^ in each plot (excluding the border ones) and adjusted to 14% moisture. The percentage of filled grains was defined as the ratio of filled grains to the total number of spikelets.

### 4.5. Statistical Analysis

The data in all the tables and figures are the means of the triplicate replicates. Data were analyzed by analysis of variance (ANOVA) with SPSS Statistics 26.0. Tukey’s test (*p* < 0.05) was used for multiple comparisons. Pearson correlation analysis and principal component analysis (PCA) were also carried out for determining the relationships between different variables. Figures were plotted using Origin 2021.

## 5. Conclusions

Controlled-release nitrogen fertilizers have gained popularity in rice cultivation due to their effectiveness in improving yield and quality with reduced environmental impact. Grain filling plays a crucial role in determining rice yield and quality, yet the effects of CRNFs on grain-filling characteristics and their relationship to rice quality remain largely unexplored. The findings of this study highlighted that an adequate carbohydrate supply during the grain-filling process, measured by a higher sugar–spikelet ratio, is essential for enhancing grain quality. A higher sugar–spikelet ratio supports improved grain filling, leading to a higher mean grain-filling rate (G_mean_), a greater maximum grain-filling rate (G_max_), and a shorter grain-filling duration (D). Compared to the CU treatment, CRNF and CRNF-CU significantly boosted the rates of brown rice, milled rice, and head milled rice, while lowering the chalky kernel rate and reducing chalkiness, thus enhancing milling and appearance quality. These treatments also increased amylose content and decreased protein content, contributing to improved grain quality. Further, CRNF and CRNF-CU treatments led to larger starch granules with smoother surfaces. They also reduced the relative crystallinity and structural order, while increasing the amorphous structure in the outer starch granule region, which lowered the stability of rice starch crystals. Additionally, these treatments raised viscosity values and enhanced the thermodynamic properties of starch, leading to better cooking and eating qualities of the rice. In summary, the CRNF-CU is the most effective strategy in this study to enhance both grain yield and quality. This approach ensures an adequate carbohydrate supply for grain filling, which is crucial for optimizing grain-filling characteristics and improving overall grain quality.

## Figures and Tables

**Table 1:** Effects of different nitrogen fertilizer treatments on grain-filling characteristics and the sugar–spikelet ratio in year 2022.

Treatment	Grain Weight (mg Grain^−1^)	G_max_ (mg Grain^−1^ d^−1^)	G_mean_ (mg Grain^−1^ d^−1^)	D (d)	Sugar–Spikelet Ratio (mg Spikelet^−1^)
CK	25.87 a	1.21 a	0.79 a	34.00 d	7.47 a
CU	24.08 c	1.04 c	0.66 c	37.11 a	5.74 d
CRNF	24.28 b	1.06 bc	0.68 bc	36.26 b	5.91 c
CRNF-CU	24.48 b	1.11 b	0.71 b	35.35 c	6.10 b

Different lowercase letters within the same column indicate significant differences at *p* < 0.05. G_max_, maximum grain-filling rate; G_mean_, mean grain-filling rate; D, active grain-filling period; CK, control check treatment; CU, conventional urea treatment; CRNF, controlled-release nitrogen fertilizer treatment; CRNF-CU, combined 30% conventional urea with 70% controlled-release nitrogen fertilizer treatment.

**Table 2:** Effects of different nitrogen fertilizer treatments on rice appearance and milling qualities.

Year	Treatment	Appearance Quality		Milling Quality		
		Chalky Kernel Rate (%)	Chalkiness (%)	Brown Rice Rate (%)	Milled Rice Rate (%)	Head Rice Rate (%)
2021	CK	20.03 c	5.21 c	82.47 b	72.29 b	69.48 b
	CU	25.13 a	6.42 a	84.07 a	74.87 a	71.48 a
	CRNF	23.23 b	5.99 b	83.60 a	73.83 a	71.37 a
	CRNF-CU	23.03 b	5.85 b	83.47 a	73.73 a	71.30 a
2022	CK	21.68 c	5.90 c	77.54 b	73.03 b	68.85 b
	CU	25.89 a	7.33 a	80.54 a	74.99 a	70.10 a
	CRNF	24.20 b	6.67 b	79.85 a	73.96 a	69.75 a
	CRNF-CU	23.98 b	6.60 b	79.66 a	73.59 a	69.68 a
Analysis of variance						
Year (Y)		**	**	**	**	**
Treatment (T)		**	**	**	**	**
Y × T		**	**	NS	NS	NS

Different lowercase letters within the same column of the same year indicate significant differences at *p* < 0.05. For analysis of variance, **, significant differences at *p* < 0.01; NS, no significant difference. CK, control check treatment; CU, conventional urea treatment; CRNF, controlled-release nitrogen fertilizer treatment; CRNF-CU, combined 30% conventional urea with 70% controlled-release nitrogen fertilizer treatment.

**Table 3:** Effects of different nitrogen fertilizer treatments on rice eating quality.

Year	Treatment	Amylose Content (%)	Amylopectin Content (%)	Total Starch Content (%)	Protein Content (%)	Gel Consistency (mm)	Hardness	Viscosity	Taste Value
2021	CK	14.22 a	55.82 a	70.04 a	7.36 b	79.65 a	5.73 c	6.30 a	75.10 a
	CU	12.67 b	52.48 b	65.15 c	9.21 a	75.52 c	6.67 a	4.80 c	58.37 c
	CRNF	13.15 b	52.98 b	66.13 b	9.11 a	76.34 b	6.40 ab	5.20 b	62.80 b
	CRNF-CU	13.40 b	53.07 b	66.47 b	8.66 a	76.64 b	6.23 b	5.33 b	63.03 b
2022	CK	14.02 a	55.64 a	69.65 a	7.45 b	79.86 a	5.87 b	6.08 a	66.40 a
	CU	12.22 c	52.12 b	64.34 c	9.32 a	72.91 c	6.83 a	4.59 c	50.73 c
	CRNF	12.93 b	52.51 b	65.44 b	9.14 a	74.10 b	6.50 a	5.01 b	56.23 b
	CRNF-CU	13.16 b	52.83 b	65.99 b	8.96 a	74.66 b	6.40 ab	5.06 b	57.57 b
Analysis of variance									
Year (Y)		*	NS	**	**	**	NS	**	**
Treatment (T)		**	**	**	**	**	**	**	**
Y × T		NS	NS	NS	NS	**	NS	NS	NS

Different lowercase letters within the same column of the same year indicate significant differences at *p* < 0.05. For analysis of variance, * and **, significant differences at *p* < 0.05 and *p* < 0.01, respectively; NS, no significant difference. CK, control check treatment; CU, conventional urea treatment; CRNF, controlled-release nitrogen fertilizer treatment; CRNF-CU, combined 30% conventional urea with 70% controlled-release nitrogen fertilizer treatment.

**Table 4:** Effects of different nitrogen fertilizer treatments on starch crystal structure of rice.

Year	Treatment	Infrared Ratio		Relatively Crystallinity (%)
		1045/1022 cm^−1^	1022/995 cm^−1^	
2021	CK	0.61 b	1.18 a	24.05 c
	CU	0.71 a	1.02 c	26.64 a
	CRNF	0.68 a	1.09 b	25.40 b
	CRNF-CU	0.67 a	1.10 b	25.31 b
2022	CK	0.60 b	1.18 a	23.45 c
	CU	0.72 a	1.02 c	26.67 a
	CRNF	0.69 a	1.10 b	25.86 b
	CRNF-CU	0.68 a	1.11 b	25.70 b
Analysis of variance				
Year (Y)		NS	NS	NS
Treatment (T)		**	**	**
Y × T		NS	NS	NS

Different lowercase letters within the same column of the same year indicate significant differences at *p* < 0.05. For analysis of variance, **, significant differences at *p* < 0.01; NS, no significant difference. CK, control check treatment; CU, conventional urea treatment; CRNF, controlled-release nitrogen fertilizer treatment; CRNF-CU, combined 30% conventional urea with 70% controlled-release nitrogen fertilizer treatment.

**Table 5:** Effects of different nitrogen fertilizer treatments on the pasting characteristics of starch.

Year	Treatment	Peak Viscosity (cP)	Hot Viscosity (cP)	Breakdown (cP)	Final Viscosity (cP)	Setback (cP)	Pasting Temperature (°C)
2021	CK	2916.41 a	1802.30 a	1114.11 a	2084.98 a	−831.44 c	73.63 c
	CU	2482.87 c	1602.60 b	880.27 c	1890.14 c	−592.73 a	75.60 a
	CRNF	2598.67 b	1659.34 b	939.33 b	1924.41 bc	−674.26 b	75.17 b
	CRNF-CU	2622.44 b	1668.03 b	954.41 b	1944.83 b	−677.61 b	75.10 b
2022	CK	2907.00 a	1794.33 a	1112.67 a	2074.67 a	−832.33 c	74.42 b
	CU	2477.00 c	1601.00 b	876.00 b	1822.67 b	−654.33 a	75.73 a
	CRNF	2585.33 bc	1657.67 b	927.67 b	1853.67 b	−731.67 b	75.18 a
	CRNF-CU	2600.33 b	1663.33 b	937.00 b	1858.00 b	−742.33 b	75.20 a
Analysis of variance							
Year (Y)		NS	NS	NS	**	**	**
Treatment (T)		**	**	**	**	**	**
Y × T		NS	NS	NS	NS	NS	**

Different lowercase letters within the same column of the same year indicate significant differences at *p* < 0.05. For analysis of variance, **, significant differences at *p* < 0.01; NS, no significant difference. CK, control check treatment; CU, conventional urea treatment; CRNF, controlled-release nitrogen fertilizer treatment; CRNF-CU, combined 30% conventional urea with 70% controlled-release nitrogen fertilizer treatment.

**Table 6:** Effects of different nitrogen fertilizer treatments on the thermal properties of starch.

Year	Treatment	*T*_O_ (°C)	*T*_P_ (°C)	*T*_C_ (°C)	Δ*H*_gel_ (J·g^−1^)
2021	CK	60.21 c	67.33 b	76.16 b	10.24 c
	CU	61.88 a	68.23 a	77.28 a	12.87 a
	CRNF	61.03 b	67.87 ab	76.74 ab	11.81 b
	CRNF-CU	60.76 bc	67.70 ab	76.60 ab	11.46 b
2022	CK	60.20 b	68.23 b	76.77 b	10.37 c
	CU	61.63 a	69.27 a	78.03 a	13.23 a
	CRNF	60.83 b	68.87 ab	77.33 b	12.39 b
	CRNF-CU	60.70 b	68.60 ab	77.23 b	12.22 b
Analysis of variance					
Year (Y)		NS	*	**	**
Treatment (T)		**	**	**	**
Y × T		NS	NS	NS	NS

Different lowercase letters within the same column of the same year indicate significant differences at *p* < 0.05. For analysis of variance, * and **, significant differences at *p* < 0.05 and *p* < 0.01, respectively; NS, no significant difference. CK, control check treatment; CU, conventional urea treatment; CRNF, controlled-release nitrogen fertilizer treatment; CRNF-CU, combined 30% conventional urea with 70% controlled-release nitrogen fertilizer treatment; *T*_O_, onset temperature; *T*_P_, peak temperature; *T*_C_, conclusion temperature; Δ*H*_gel_, gelatinization enthalpy.

## Data Availability

The data presented in this study are available upon request from the corresponding author.
